# Supplementary material for: Towards explicit regulating-ion-transport: nanochannels with only function-elements at outer-surface
Source: Nat Commun. 2021 Mar 10;12:1573. doi: 10.1038/s41467-021-21507-7 (PMC7946920; doi:10.1038/s41467-021-21507-7)
Supplement: Supplementary file 1 — Supplementary Information [file 41467_2021_21507_MOESM1_ESM.pdf]

Supplementary Materials For

Towards Explicit Regulating-Ion-Transport: Nanochannels with only Function-Elements at Outer-Surface

This PDF file includes:

- **Supplementary Fig. 1.** Sketch map of metallic depositions.
- **Supplementary Fig. 2.** Unilateral attachment of FE at the outermost layer of Au.
- **Supplementary Fig. 3.** Characterization of the structure of nanochannels.
- **Supplementary Fig. 4.** Energy Dispersive Spectrometer (EDS) mapping along the none@OS.
- **Supplementary Fig. 5.** Contact angle and surface zeta potential of raw AAO and none@OS.
- **Supplementary Fig. 6.** Ion transport of raw AAO and none@OS.
- **Supplementary Fig. 7.** Sketch map of the functionalization of OS.
- **Supplementary Fig. 8. The structure of functional elements used in this work.**
- **Supplementary Fig. 9.** The depth determination of functional elements along the nanochannel using ToF-SIMS and SEM.
- **Supplementary Fig. 10.** The Statistic parameter of the PAA@OS, PEI@OS and DNA@OS, respectively.
- **Supplementary Fig. 11.** Surface zeta potential at different pH of PAA@OS, PEI@OS and DNA@OS, respectively.

- **Supplementary Fig. 12.** Surface properties of OS characterized by AFM.
- **Supplementary Fig. 13.** The effect of the ion strength on the ion transport across nanochannels.
- **Supplementary Fig. 14.** Simulated model in COMSOL.
- **Supplementary Fig. 15.** The calculated ion concentration profiles.
- **Supplementary Fig. 16.** Equivalent circuit model for the nanochannel system.
- **Supplementary Fig. 17.** The electrical conductance of none@OS.
- **Supplementary Fig. 18.** I-V curves of the none@OS under two different configurations for the placement of electrolyte solutions.
- **Supplementary Fig. 19.** AFM images of none@OS functionalized with different PAA concentration.
- **Supplementary Fig. 20.** The output power density of the none@OS functionalized with different PAA concentration based osmotic power generator.
- **Supplementary Fig. 21.** I-V curves at a concentration gradient of 500 using different PAA concentration.
- **Supplementary Fig. 22.** I-V curves of nanochannel with OS functionalized by layer-by-layer assembly of polyelectrolyte, beginning with PAA.
- **Supplementary Fig. 23.** Surface zeta potential of nanochannel with OS attached with layer-by-layer assembled polyelectrolyte.
- **Supplementary Fig. 24.** The contract of f_{rec} of PAA_4PEI_3 and PAA_4PEI_4.
- **Supplementary Fig. 25.** I-V curves of nanochannel of nanochannel with OS functionalized with layer-by-layer assembly of polyelectrolyte, beginning with PEI.

- **Supplementary Fig. 26.** The effect of layer-by-layer assembly of charged polyelectrolytes at the OS of none@OS on the ion current rectification of nanochannels.
- **Supplementary Fig. 27.** The internal resistance of nanochannels with LBL polyelectrolytes as FE.
- **Supplementary Fig. 28.** The detection limit of multi-targets.
- **Supplementary Fig. 29.** Comparison of the distribution percentage of ssw-DNA at IW in the 1st and 3rd stage.
- **Supplementary Fig. 30.** Chronocoulometric quantitation of surface charge density of OS attached with sswDNA.
- **Supplementary Fig. 31.** The effect of DNA grafting density on the ion transport.
- **Supplementary Fig. 32.** The ion transport properties of nanochannels with hydrophobic molecules (DM) at OS.
- **Supplementary Fig. 33.** The reuse properties of nanochannel.
- **Supplementary Fig. 34.** The contract of reaction dynimic of nanochannel with FE_{OS} and FE_{IW}.
- **Supplementary Table S1.** The calculated area of IW and OS in previous reports.
- **Supplementary Table S2.** The DNA sequences to prepare the ssw-DNA structure.
- **Supplementary Table S3.** DNA aptamer sequences for the detection of targets.
- **Supplementary Table S4.** Boundary conditions for the numerical solution.
- **References for Supplementary Information.**

1. Supplementary Figures

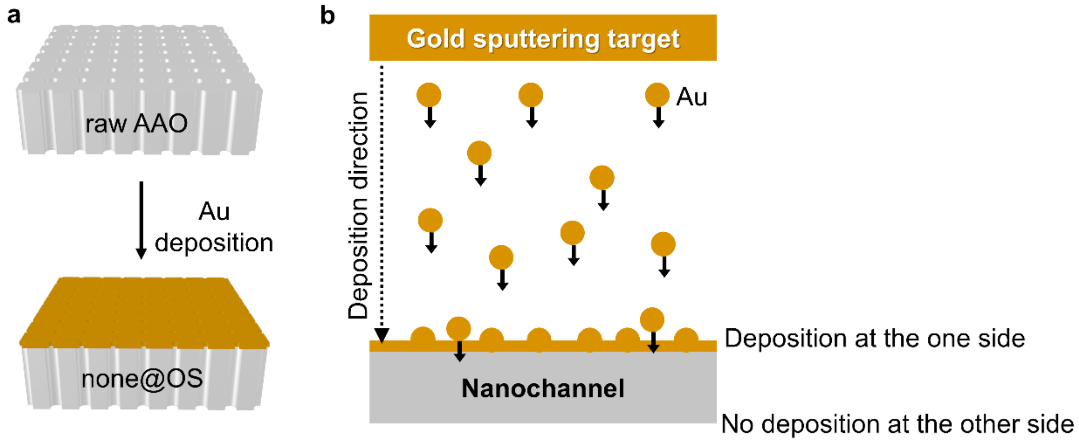

Supplementary Fig. 1. Sketch map of metallic depositions. The Au deposition perpendicular to the outmost surface of AAO membrane and at the one side of the AAO membrane's OS, and no deposition at the other side of AAO membrane.

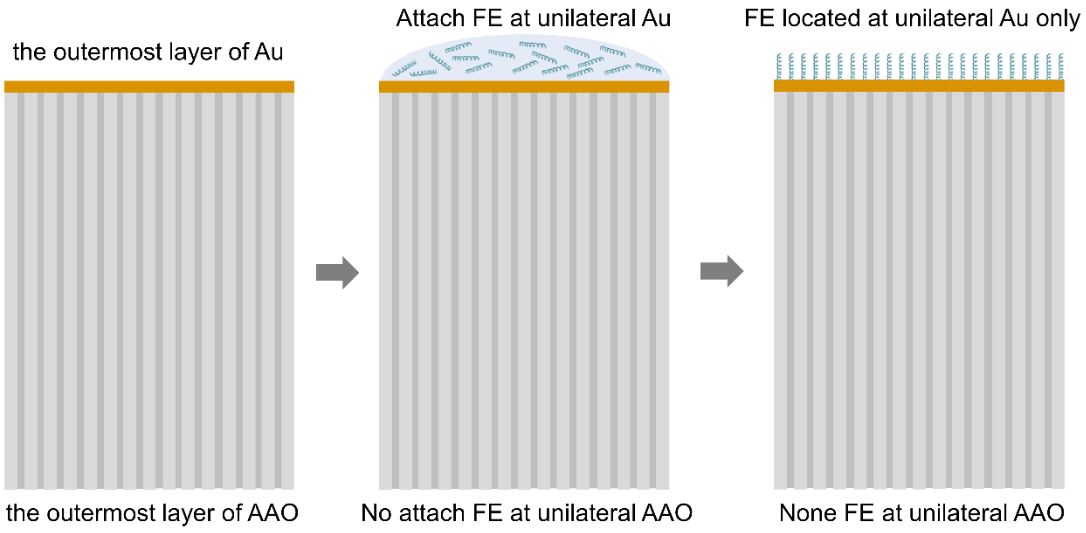

Supplementary Fig. 2. Unilateral attachment of FE at the outermost layer of Au. The FE solution were added at the outermost layer of Au. Due to the threshold effect, the FE were detained at the unilateral side of the Au layer.

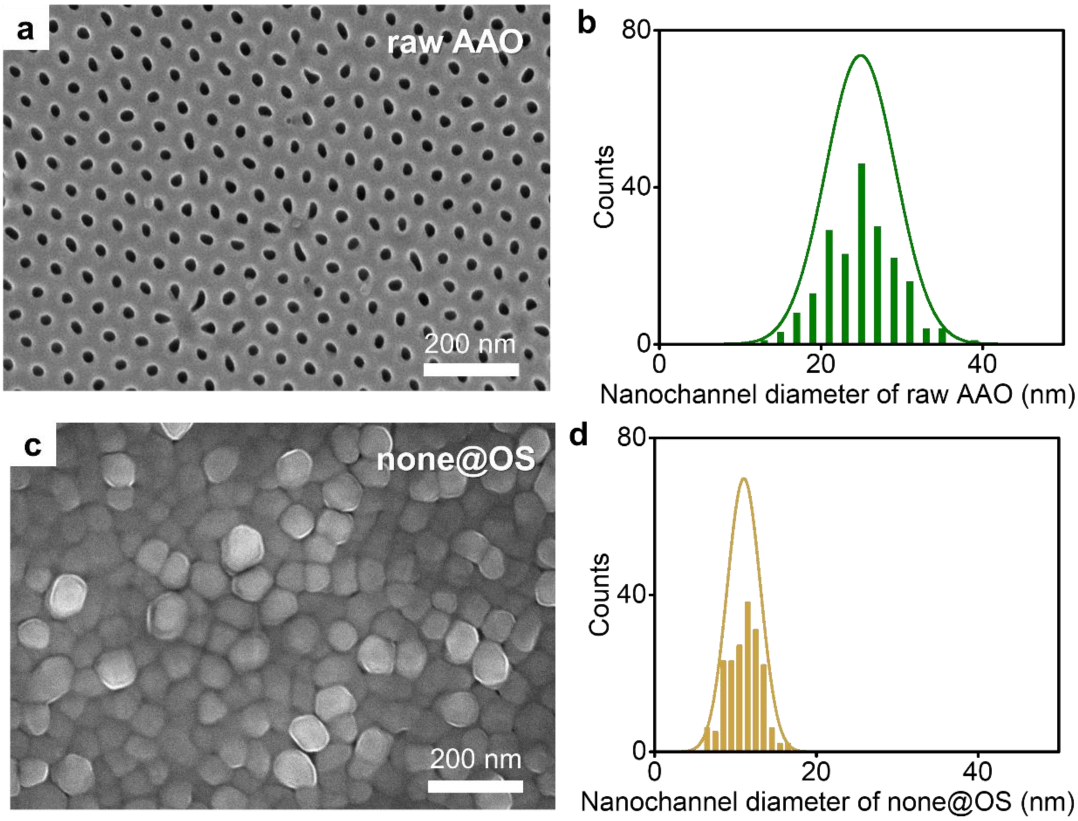

Supplementary Fig. 3. Characterization of the structure of nanochannels. (**a, c**) SEM images of before and after Au deposition. **b**, Distribution of raw AAO diameter with an average value of 25 ± 5 nm. **d**, Distribution of none@OS diameter with an average value of 11 ± 3 nm. Every sample was tested 200 dots to observe the average values.

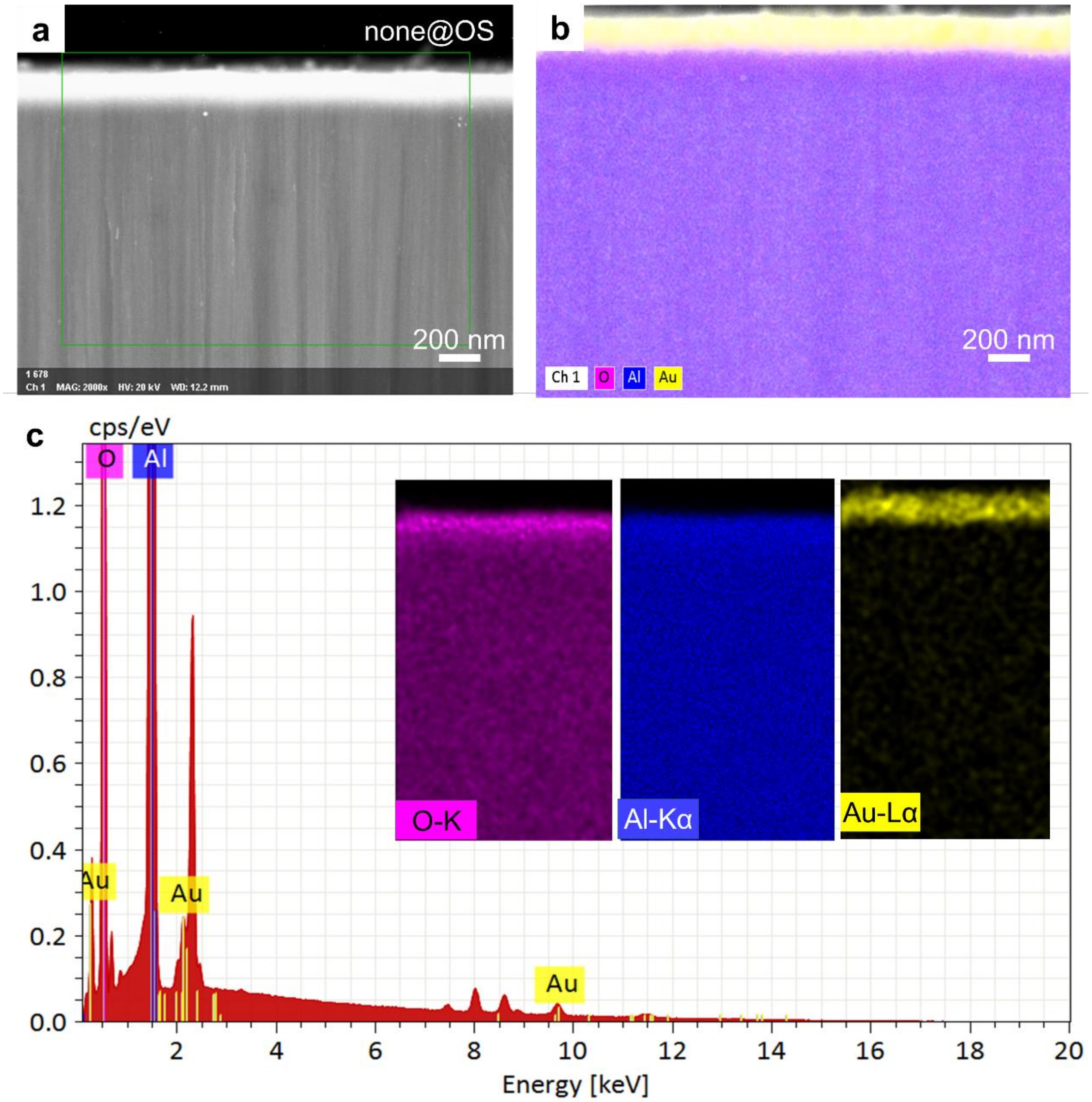

Supplementary Fig. 4. Energy Dispersive Spectrometer (EDS) mapping along the none@OS. a, SEM image of none@OS. Mapping scanning taking along the channel. (**b**, **c**) The corresponding EDS mapping of none@OS, the insets in **c** are the cross-sectional mapping images for O, Al and Au.

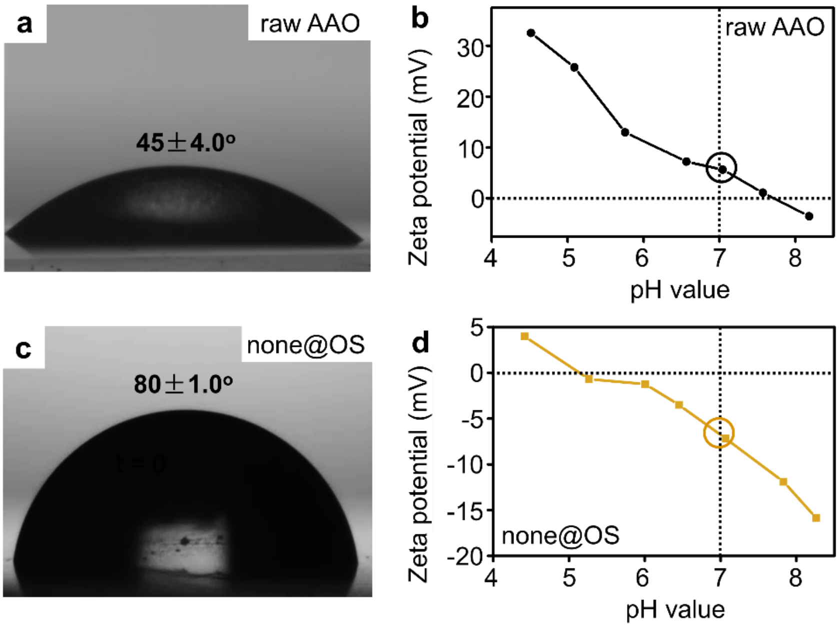

Supplementary Fig. 5. Contact angle and surface zeta potential of raw AAO and none@OS. (a, c) Contact angle (CA) of raw AAO and none@OS. After Au deposition, the CA increased from 45±4.0° to 80 ± 1.0° (The CA of raw AAO and none@OS were recorded at the 10th second, in consider of the permeation of water). (**b**, **d**) Zeta potential (ζ) at different pH of OS(Au) and OS(AAO), respectively. The tests were performed using 0.1 M KCl under different pH. The surface zeta potential varied from 5.64 mV (raw AAO) to -7.19 mV (none@OS) before and after Au deposition (pH 7). According to the Gouy-Chapmann equation, the surface charge could be calculated, respectively.

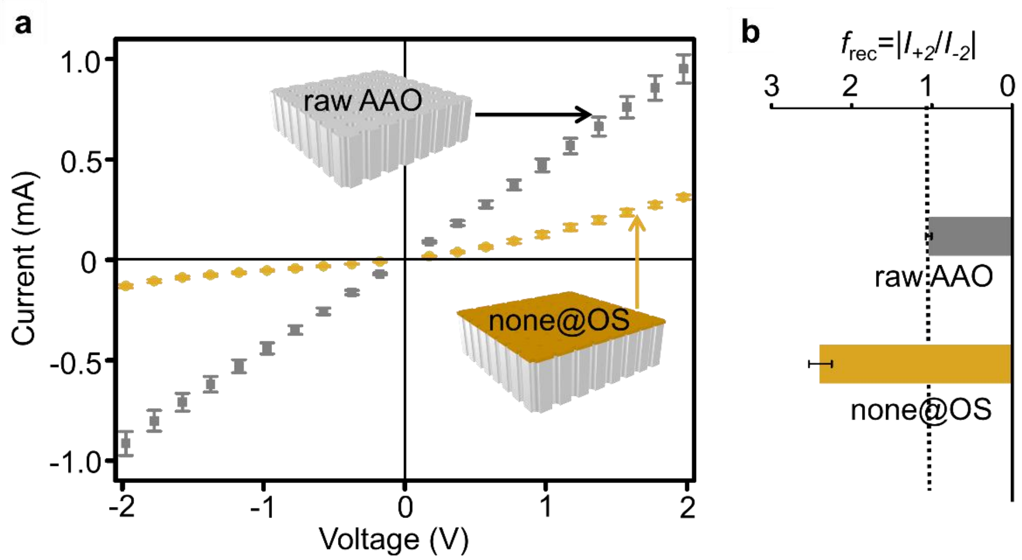

Supplementary Fig. 6. Ion transport of raw AAO and none@OS. a, I-V curves of raw AAO and none@OS measured in 0.1 M KCl solutions (pH 7.0). **b,** The ion current rectification (f_{rec}) of raw AAO and none@OS, respectively. The I-V curve exhibits different characters of raw AAO and none@OS. The curve of raw AAO is lineal, while the curve of none@OS is nolinear, a typical characteristic of ion current rectification. The f_{rec} of none@OS is 2.3. Error bars represent the standard deviation of calculations of 5 samples at least.

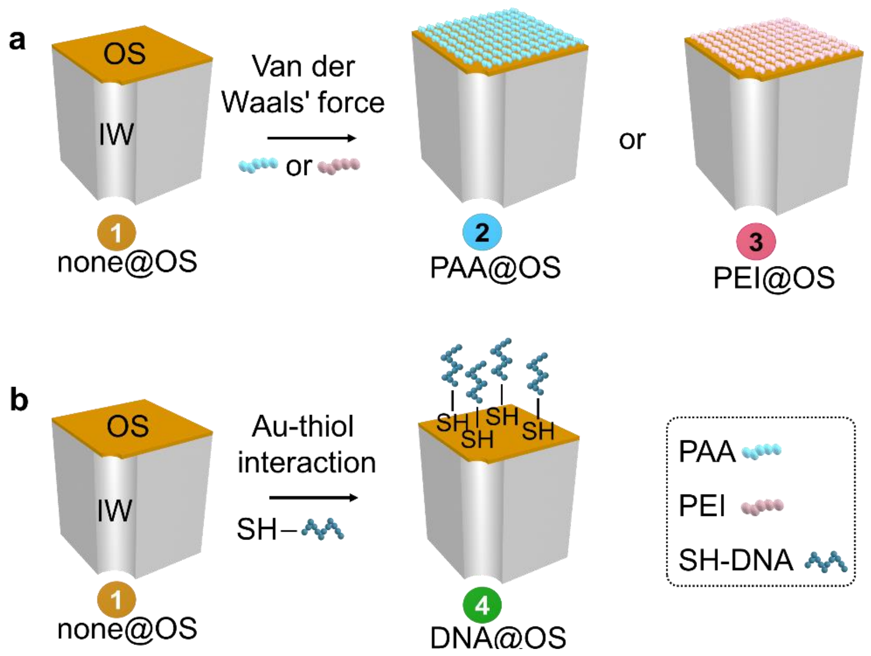

Supplementary Fig. 7. Sketch map of the functionalization of OS. a, PAA and PEI through Van der Waals' force. **b**, DNA through Au-thiol interactions.

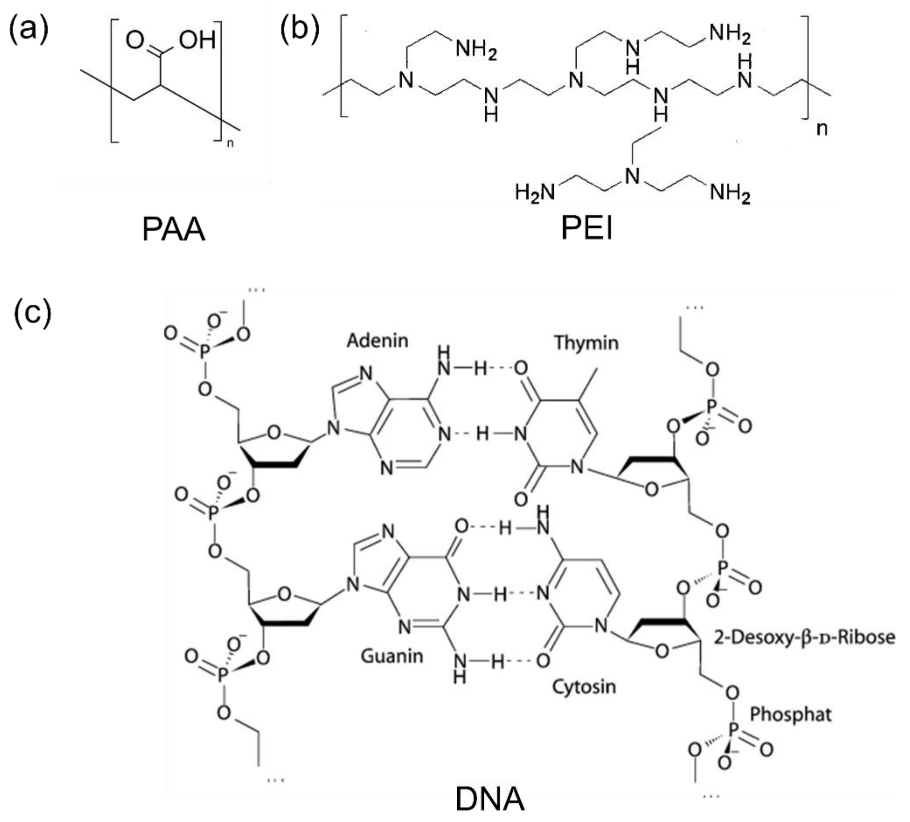

Supplementary Fig. 8. The structure of functional elements used in this work.

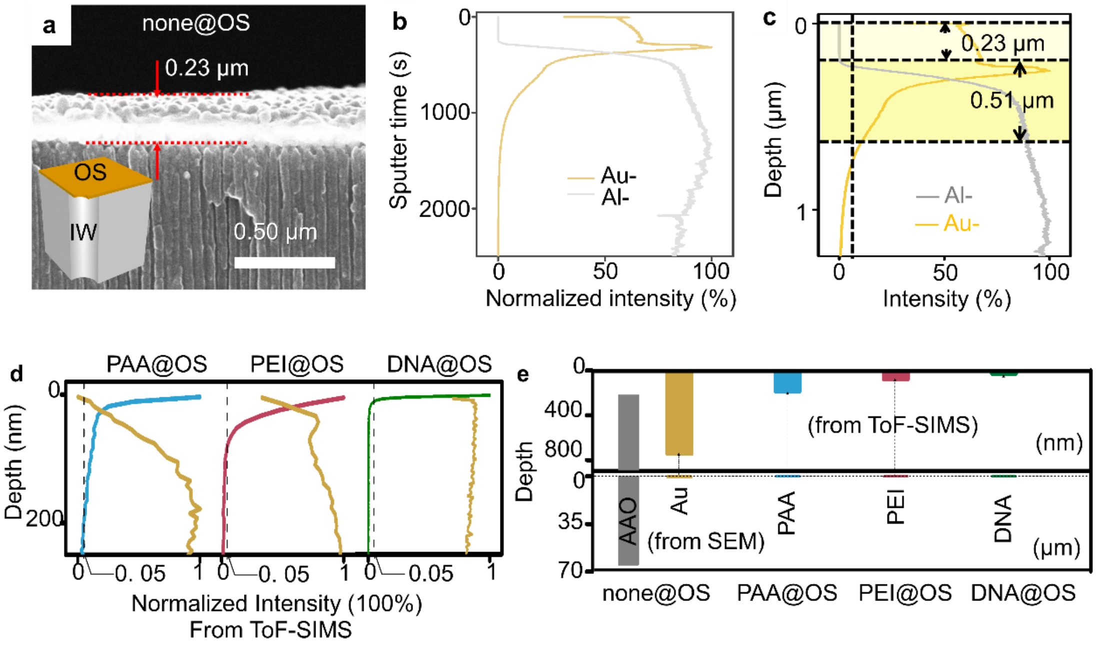

Supplementary Fig. 9. The depth determination of functional elements along the nanochannel using ToF-SIMS and SEM. a, SEM images record the sectional view of nanochannel system. **b**, The intensity records of ToF-SIMS from the outermost Au layer in none@OS. **c**, The corresponding depth distribution of functional elements along the nanochannel. If the boundary at 5 % of peak intensities is defined as the start or the end of detection signal, the depth of Au in the none@OS(Au) is 740 nm, which consists of two parts as an Au region of 230 nm with only Au^- signals and an Au region of 510 nm coexisting with Au^- and Al^- signals. It shows that the Au film is separated by two parts, as one part is at the OS while the other part is at the IW. The thickness of Au at the OS (230 nm) is far smaller than the pore height of AAO nanochannels (65 µm). **d** and **e**, depth distribution of the part of FE_{OS} in none@OS, PAA@OS, PEI@OS and DNA@OS using the ToF-SIMS and SEM.

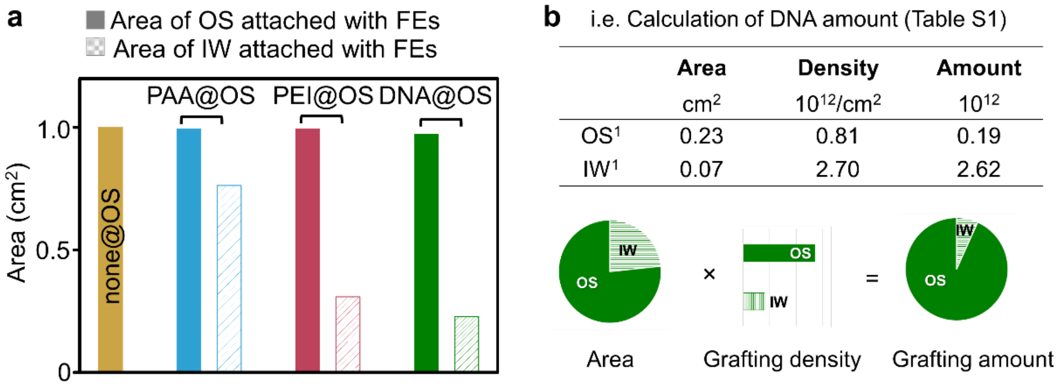

Supplementary Fig. 10. The Statistic parameter of the PAA@OS, PEI@OS and DNA@OS, respectively. a, the area of functional elements at OS and IW, respectively. **b**, the rough calculation of the grafting amount of DNA at OS. The grafting density of the FE at IW and the FE at OS comes from the literature using the DNA of same sequence[1].

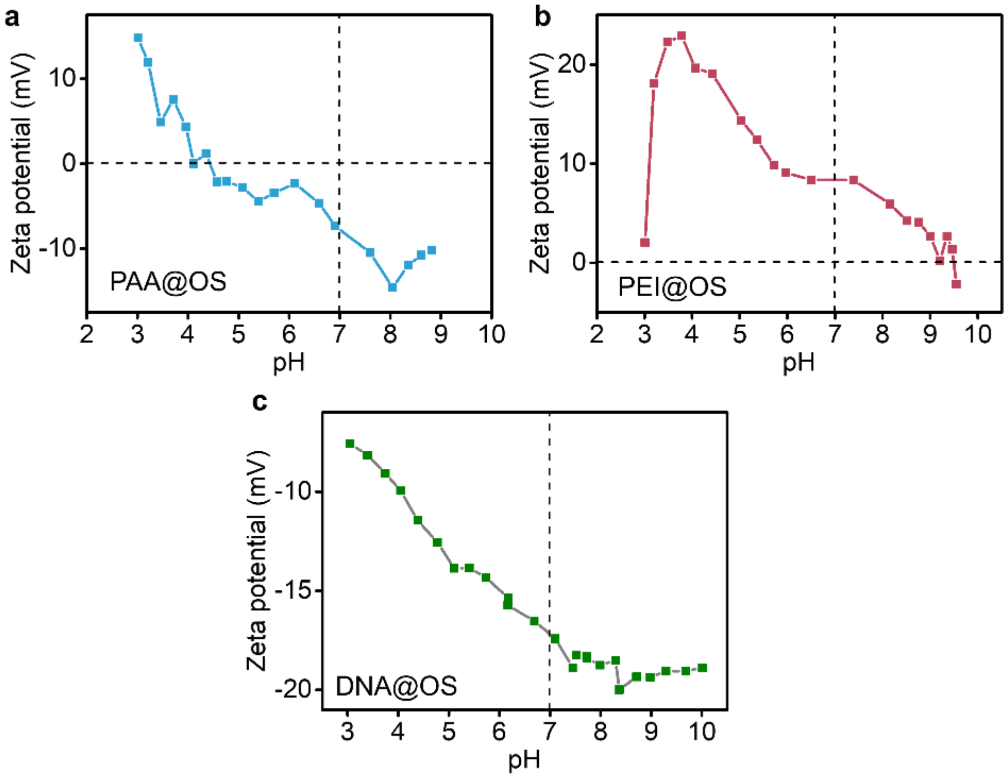

Supplementary Fig. 11. Surface zeta potential at different pH of PAA@OS, PEI@OS and DNA@OS, respectively. Accordingly, the surface charged density of the OS (σ) could be calculated based on the Gouy-Chapmann equation by measured value of zeta potential.

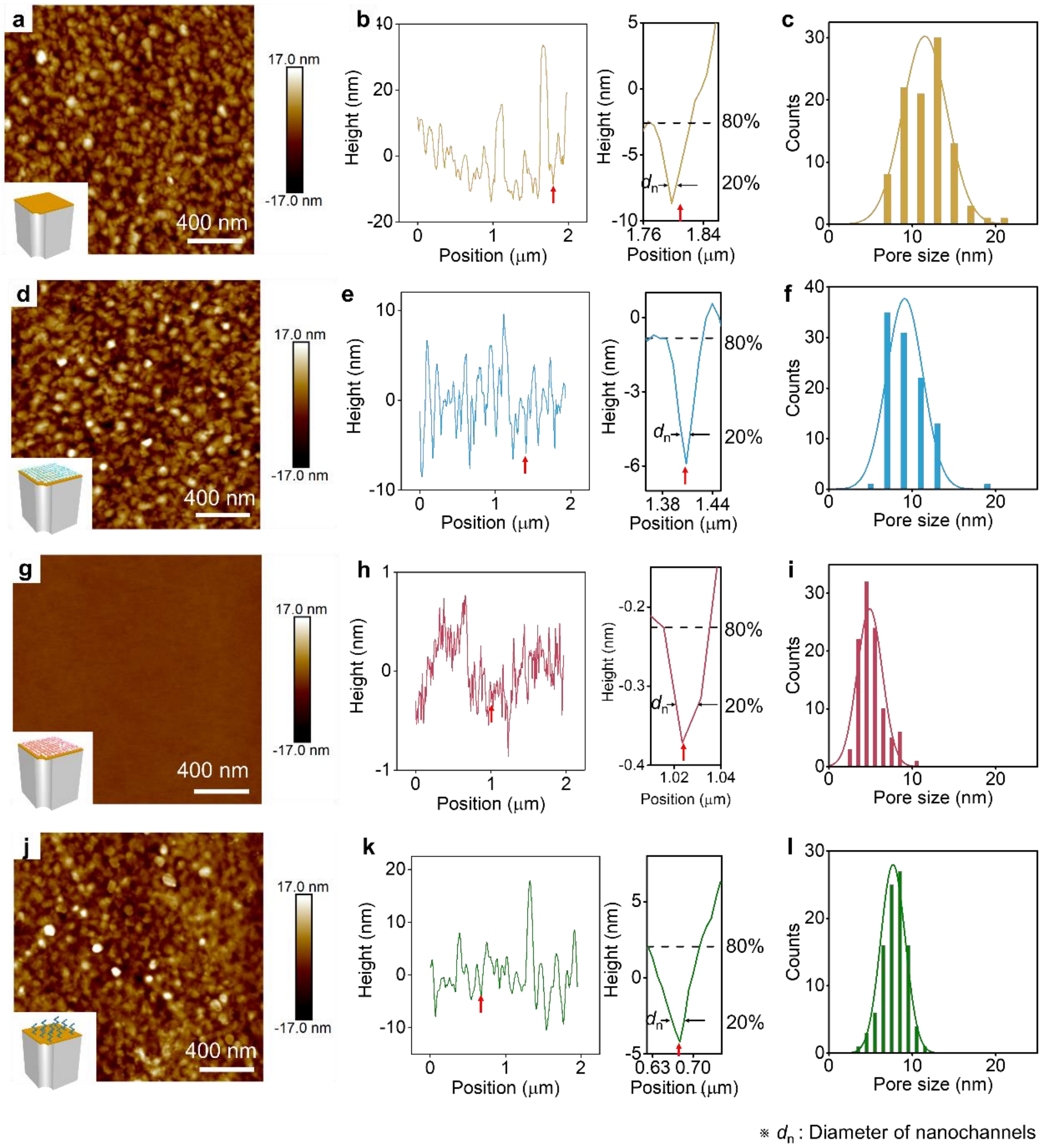

Supplementary Fig. 12. Surface properties of OS characterized by AFM. The AFM images of none@OS (**a**), PAA@OS (**d**), PEI@OS (**g**) and DNA@OS (**j**), respectively. The corresponding height versus position plots of none@OS (**b**), PAA@OS (**e**), PEI@OS (**h**) and DNA@OS (**k**), respectively. The histograms of pore size distribution of none@OS (**c**), PAA@OS (**f**), PEI@OS (**i**) and DNA@OS (**l**), respectively. Here, we used the width of peak valley with 80% intensity as pore size according the plots of height vs. position. Each sample was tested 100 dots to obtain the average values.

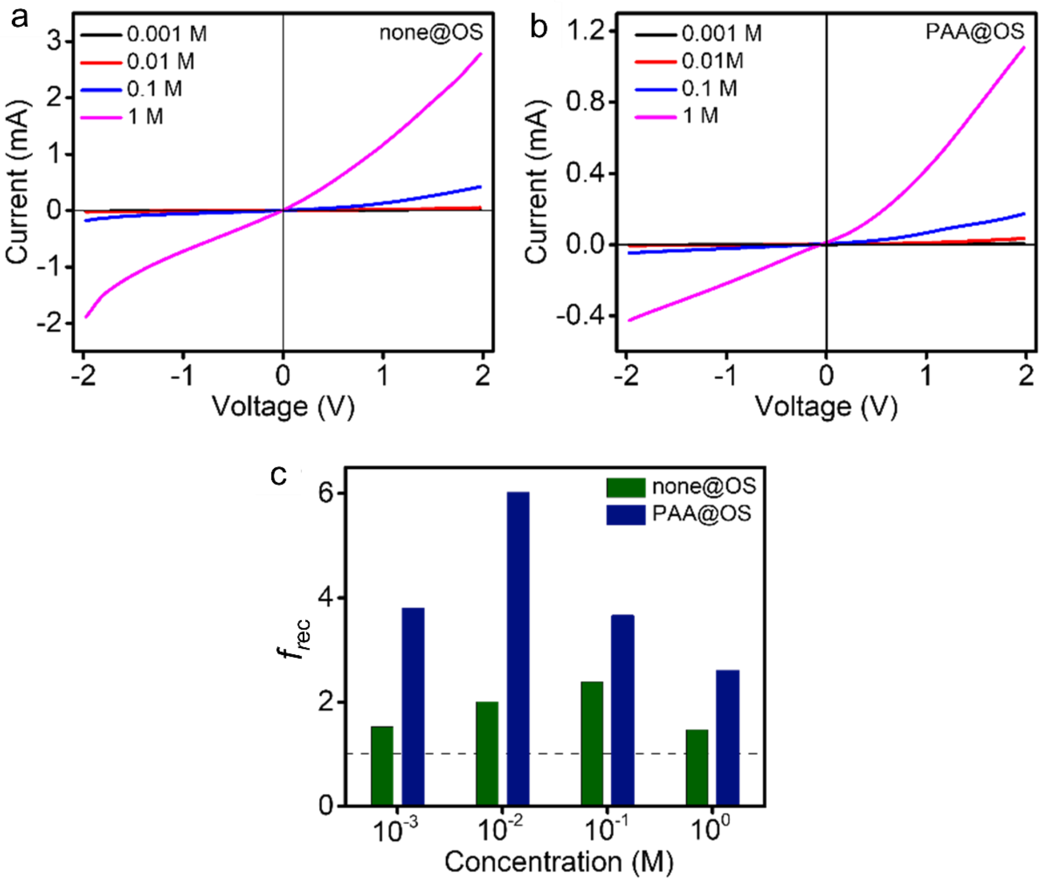

Supplementary Fig. 13. The effect of the ion strength on the ion transport across nanochannels.

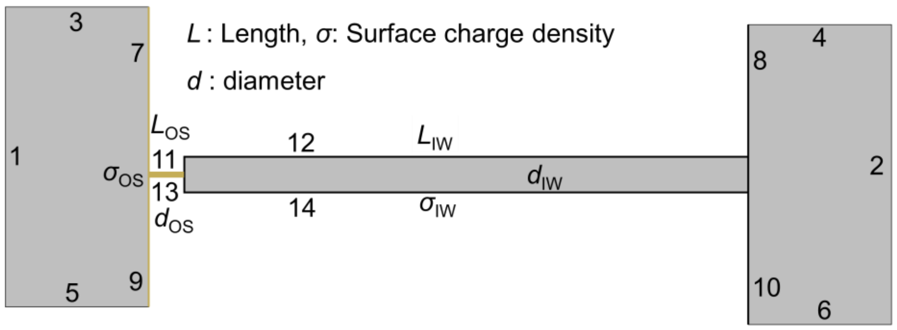

Supplementary Fig. 14. Simulated model in COMSOL. The model described the geometric dimension of nanochannels and the charge distribution long the nanochannels, which are valued using the measured parameters to analyze the effect of FE_{OS} on the ion transport of nanochannel system. The total length of nanochannel, FE and Au were scaled down at the same proportion based on the measured parameters due to the limitation of simulations. Two reservoirs were set to keep the system stable. In the simulation, the pore diameter was set to be 25 nm and the pore diameter for none@OS, PAA@OS, PEI@OS and DNA@OS side was used according to the measured parameters. An adaptive mesh refinement was used to optimize the mesh size geometry. The boundary conditions for eqs 1 to 3 are summarized in Table S4, using the numbers 1-14 to designate the surfaces defining the model system.

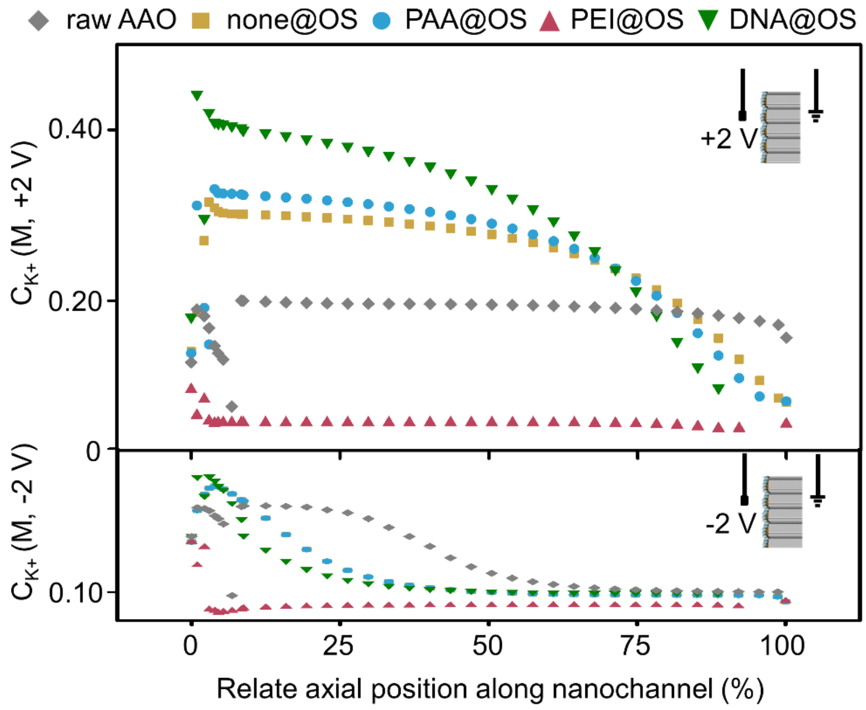

Supplementary Fig. 15. The calculated ion concentration profiles. The concentration distribution file along axial variations for individual ions at +2 V (**a**) and -2 V (**b**) based on the numerical simulation.

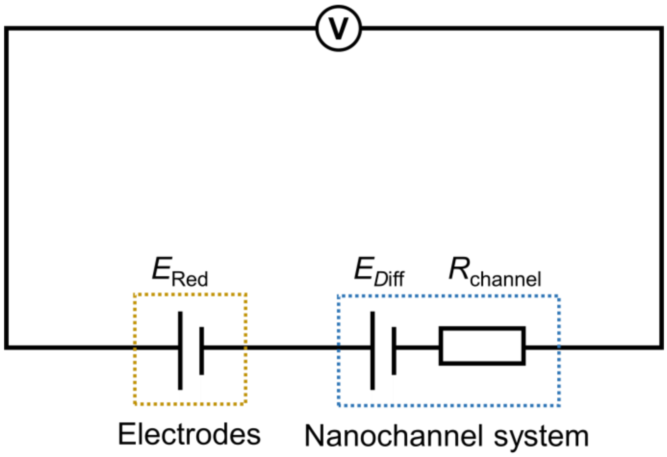

Supplementary Fig. 16. Equivalent circuit model for the nanochannel system. E_{Mea}, E_{Red}, E_{Diff}, and R_{channel} are the measured potential, the potential generated from redox reactions on the electrodes, the diffusion potential of the nanochannels, and the internal resistance of the nanochannels, respectively. Under a concentration gradient, a potential drop is generated by the redox reaction on the electrode/electrolyte interface (E_{Red}). But only the diffusion potential (E_{Diff}) is contributed from the cation-selective PAA@OS. According to the previous works[2], E_{Diff} is calculated as $E_{\text{Diff}} = E_{\text{Mea}} - E_{\text{Red}}$.

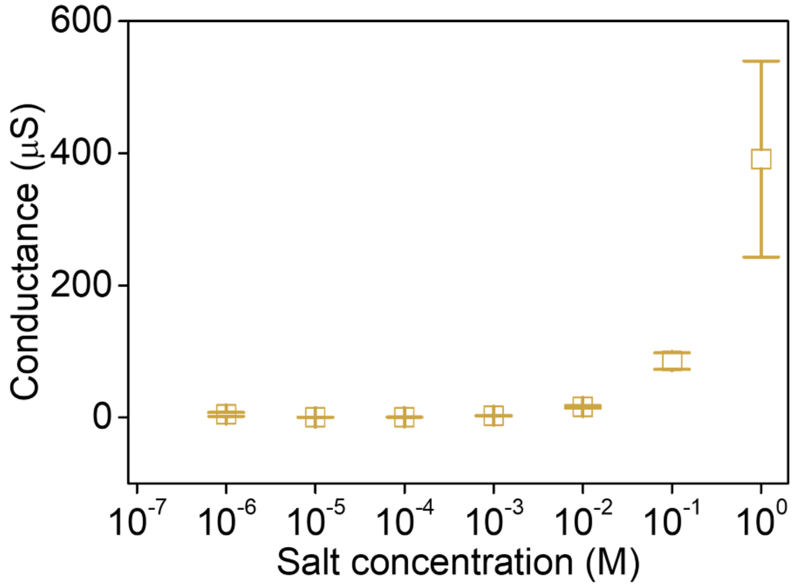

Supplementary Fig. 17. The electrical conductance of none@OS. Conductance versus salt concentration for none@OS at pH 7. The conductance depends on the salt concentration and shows saturation at low salt concentrations, indicating a signature of the presence of surface charge on the nanochannel system. Error bars represent the standard deviation of calculations of 3 samples at least.

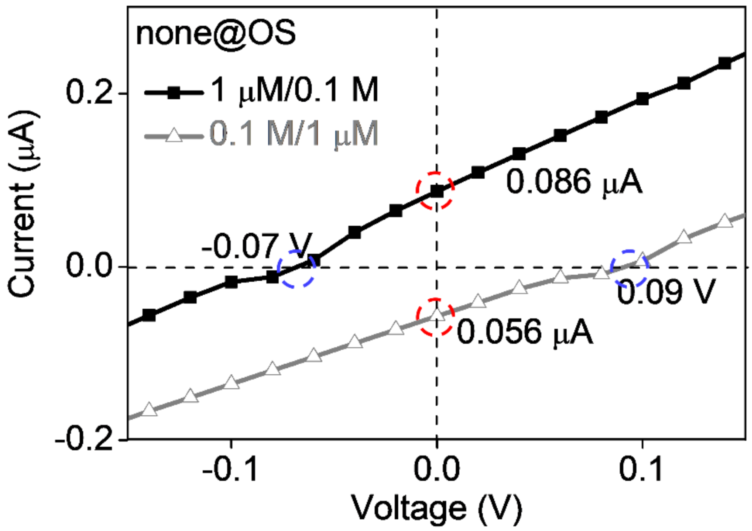

Supplementary Fig. 18. I-V curves of the none@OS under two different configurations for the placement of electrolyte solutions. With the high concentration of KCl solution on the AAO side, the absolute value of I short increases by approximately 35%. The high and low concentrations of KCl solutions are 0.1 M and 1 μM, respectively. The enhanced ion diffusion from Au side toward AAO side reduces the internal resistance of the nanochannel system ($R_{channel}$) by nearly 53%. Hence, in the following tests, the concentration on the Au side was kept high.

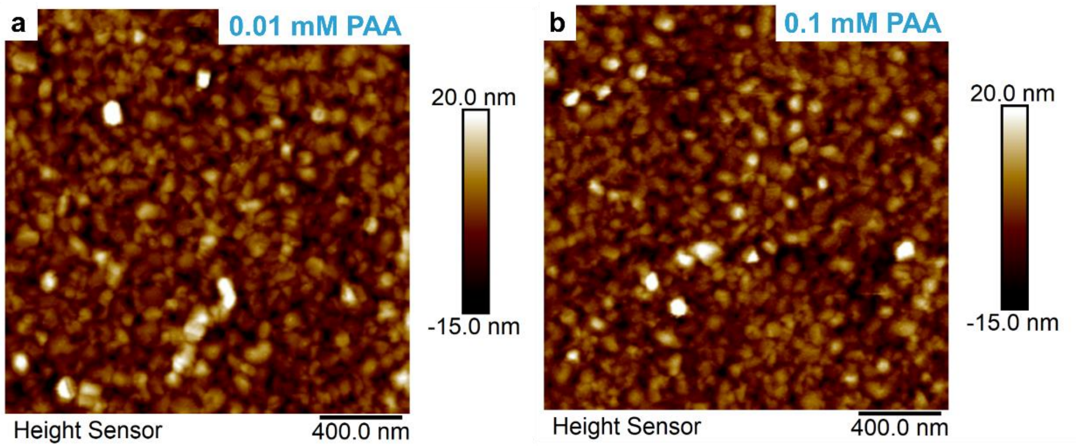

Supplementary Fig. 19. AFM images of none@OS functionalized with different PAA concentration. a, 0.01 mM PAA. **b**, 0.01 mM PAA.

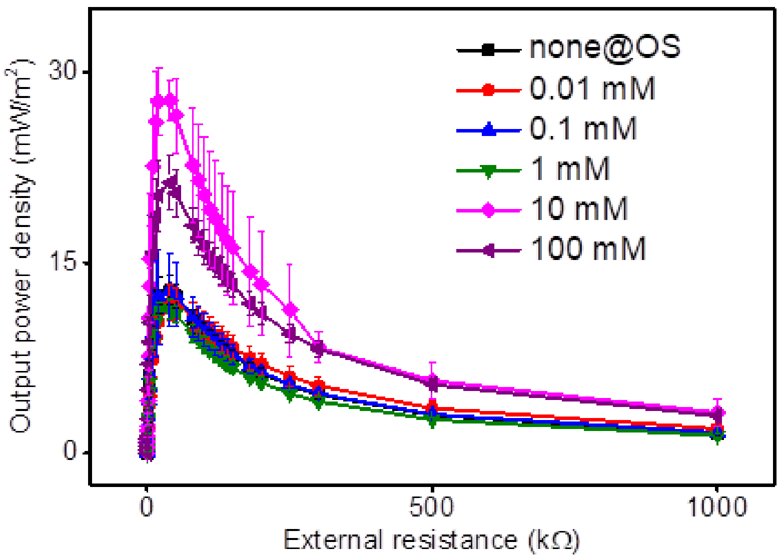

Supplementary Fig. 20. The output power density of the none@OS functionalized with different PAA concentration based osmotic power generator. Output power of none@OS and PAA@OS as functions of load resistance. The Au side was placed to artificial seawater (5 M NaCl), while the AAO side was placed to artificial river water (0.01 M NaCl). With increasing load resistance, the output power reaches a maximum at a moderate resistance. Error bars represent the standard deviation of calculations of 3 samples at least.

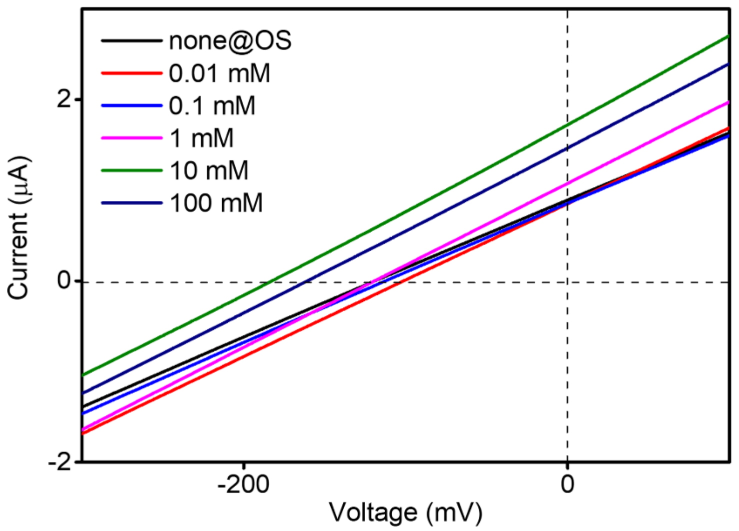

Supplementary Fig. 21. I-V curves at a concentration gradient of 500 using different PAA concentration. The contribution from the redox reaction on the electrodes is not subtracted from the measured total current. Using this result, the ion selectivity can be obtained based on the equation as below[2]:

$$2t_+ - 1 = \frac{E_{\text{diff}}}{\frac{RT}{zF}\ln\left(\frac{\gamma_{c_H} c_H}{\gamma_{c_L} c_L}\right)}$$

where R, T, z, F, γ, c_H, and c_L represent the gas constant, temperature, charge valence, Faraday constant, activity coefficient of ions, high and low ion concentrations, respectively. The t_+ represents the transference number for cations, which quantifies the selective ion transportation through nanochannel systems.

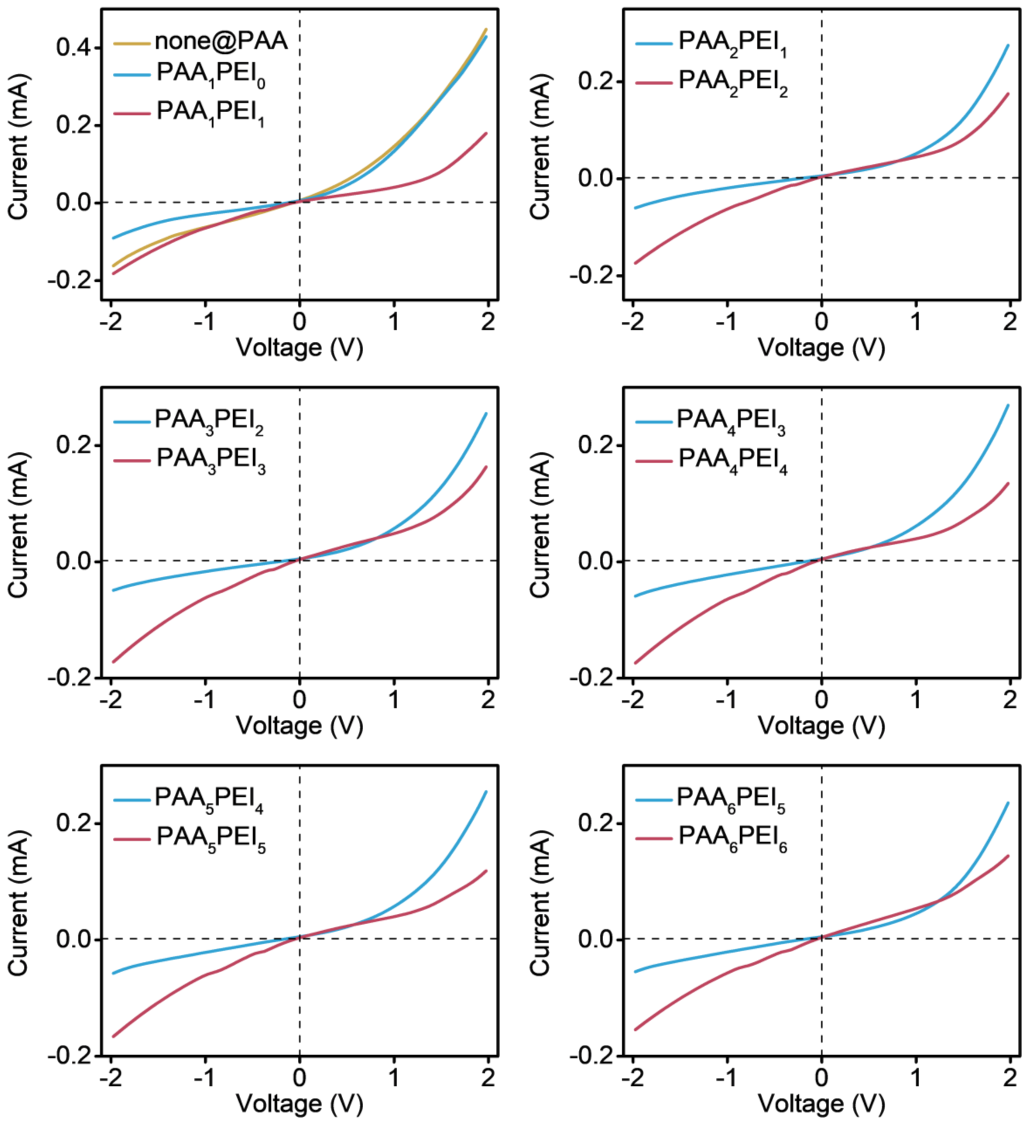

Supplementary Fig. 22. *I-V* curves of nanochannel with OS functionalized by layer-by-layer assembly of polyelectrolyte, beginning with PAA. Here, positive-charged PEI and negative-charge PAA overlapped layer-by-layer on the OS of none@OS through electrostatic assembly. The concentration of PEI and PAA solution is the same as 1 mM for all coating in following layer-by-layer experiments. The numbers in sample names descripts the coating number of times. For instance, PAA_3PEI_2 means three times PAA coating and two times PEI coating, beginning with PAA coating

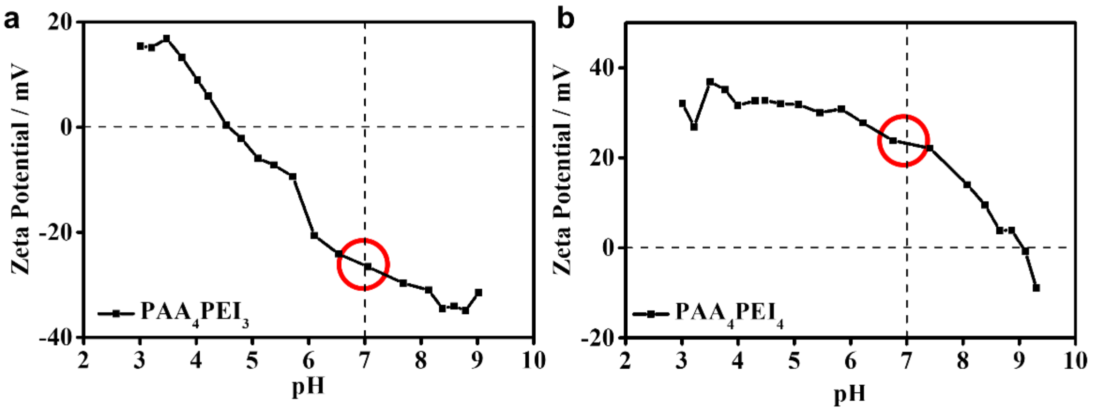

Supplementary Fig. 23. Surface zeta potential of nanochannel with OS attached with layer-by-layer assembled polyelectrolyte. a, PAA_4PEI_3. **b**, PAA_4PEI_4.

a

	Depth (nm)	Diameter (nm)	Charge (mC/m^2)
PAA_4PEI_3	10	~1.0	-5.8
PAA_4PEI_4	20	~1.0	6.1

b

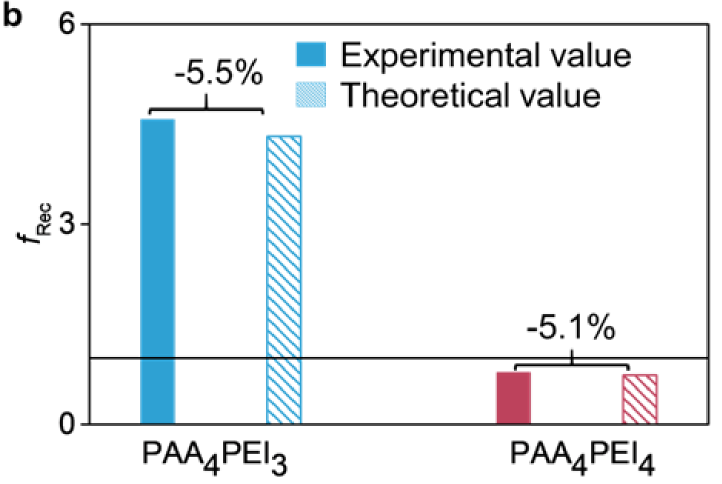

Supplementary Fig. 24. The contract of f_{rec} of PAA4PEI3 and PAA4PEI4. a, the measured data. **b**, the experimental f_{rec} of PAA4PEI3 and PAA4PEI4 and the theoretical f_{rec} using the measured data.

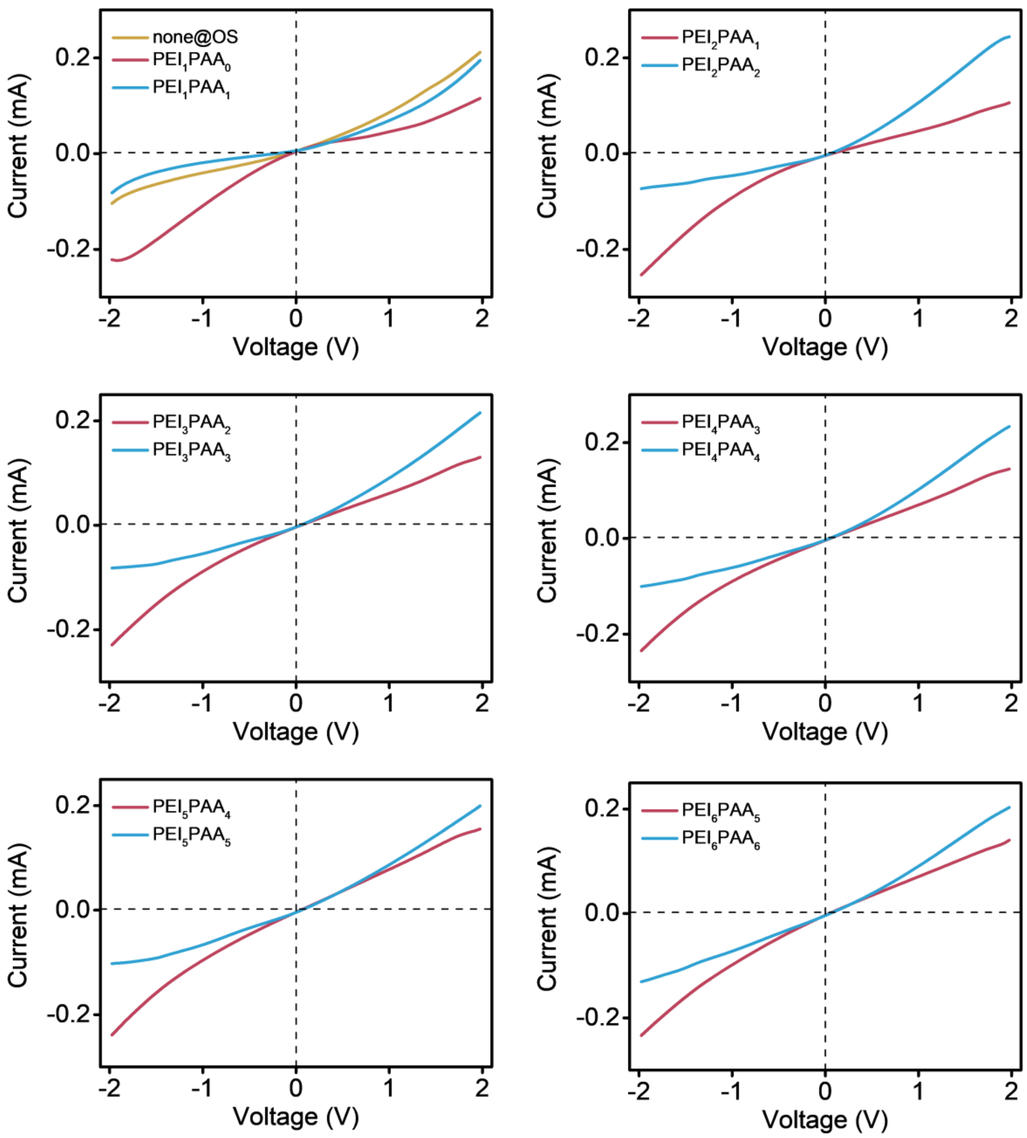

Supplementary Fig. 25. *I-V* curves of nanochannel of nanochannel with OS functionalized with layer-by-layer assembly of polyelectrolyte, beginning with PEI. In this case, when initially coating a PEI layer at OS, the forward direction reverses from negative to positive compared with none@OS. The sequent PAA coating causes the reverse of ICR direction. The reverse of ICR direction with nearly no decrease of ICR ratio can also keep for 6 cycles.

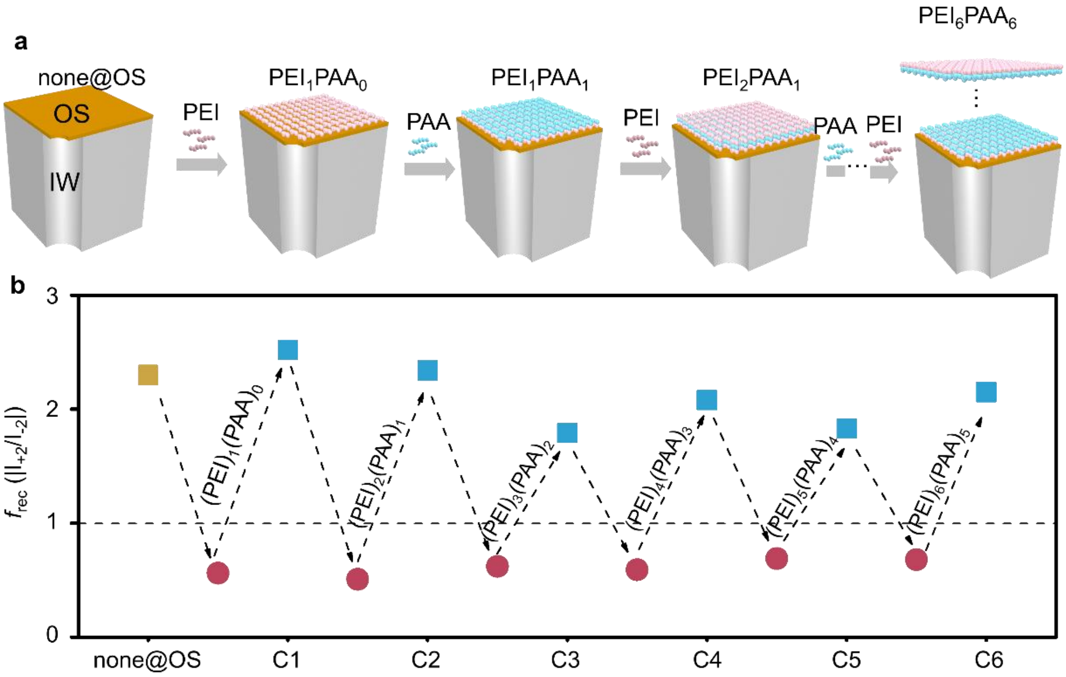

Supplementary Fig. 26. The effect of layer-by-layer assembly of charged polyelectrolytes at the OS of none@OS on the ion current rectification of nanochannels. a, Schematic illustration of the sequential (PEI)(PAA) assembly starting with PEI polyelectrolyte. **b**, the corresponding f_{rec} as a function of the number of layers in the polyelectrolyte assembly.

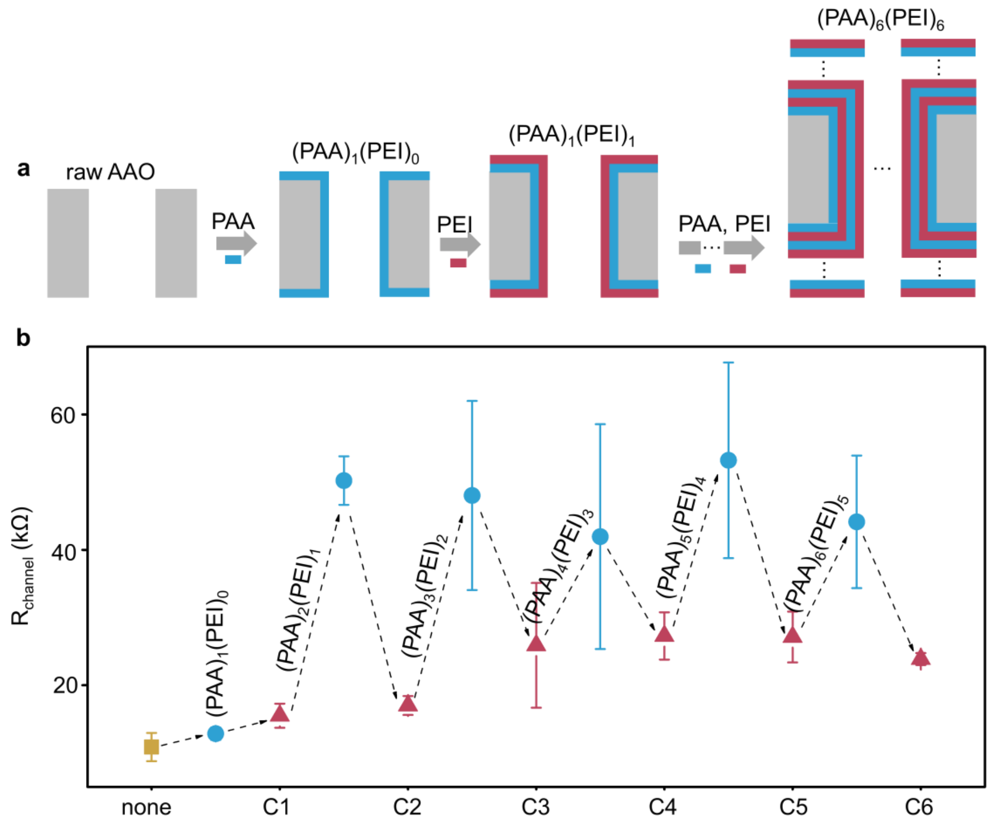

Supplementary Fig. 27. The internal resistance of nanochannels with LBL polyelectrolytes as FE. The internal resistance obviously increased when the nanochannel was functionalized with LBL polyelectrolytes. Error bars represent the standard deviation of calculations of 3 samples at least.

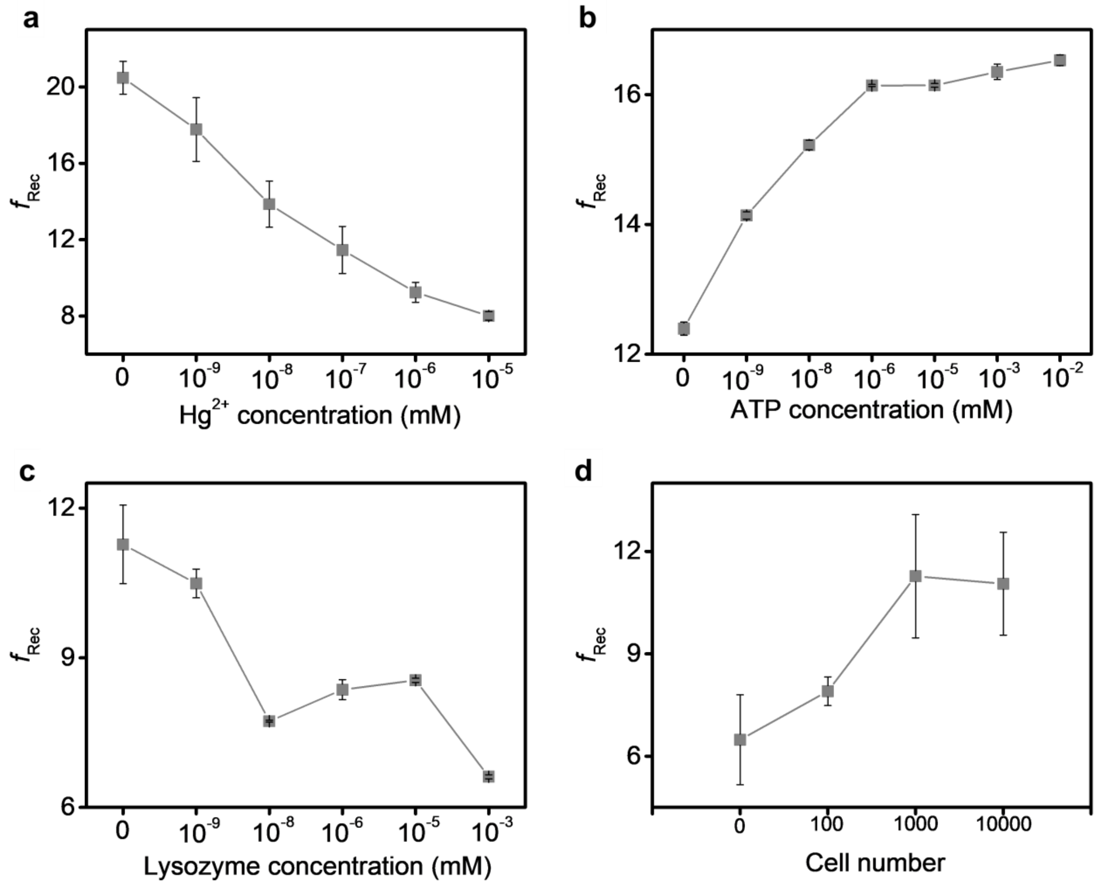

Supplementary Fig. 28. The detection limit of multi-targets. a, Hg^{2+}, **b,** ATP, **c,** Lysozyme, and **d,** MCF-7 cells using nanochannels attached with designed single-stranded DNA at OS (acting as FE_{OS}) having specific combination force with targets. Error bars represent the standard deviation of calculations of 5 samples.

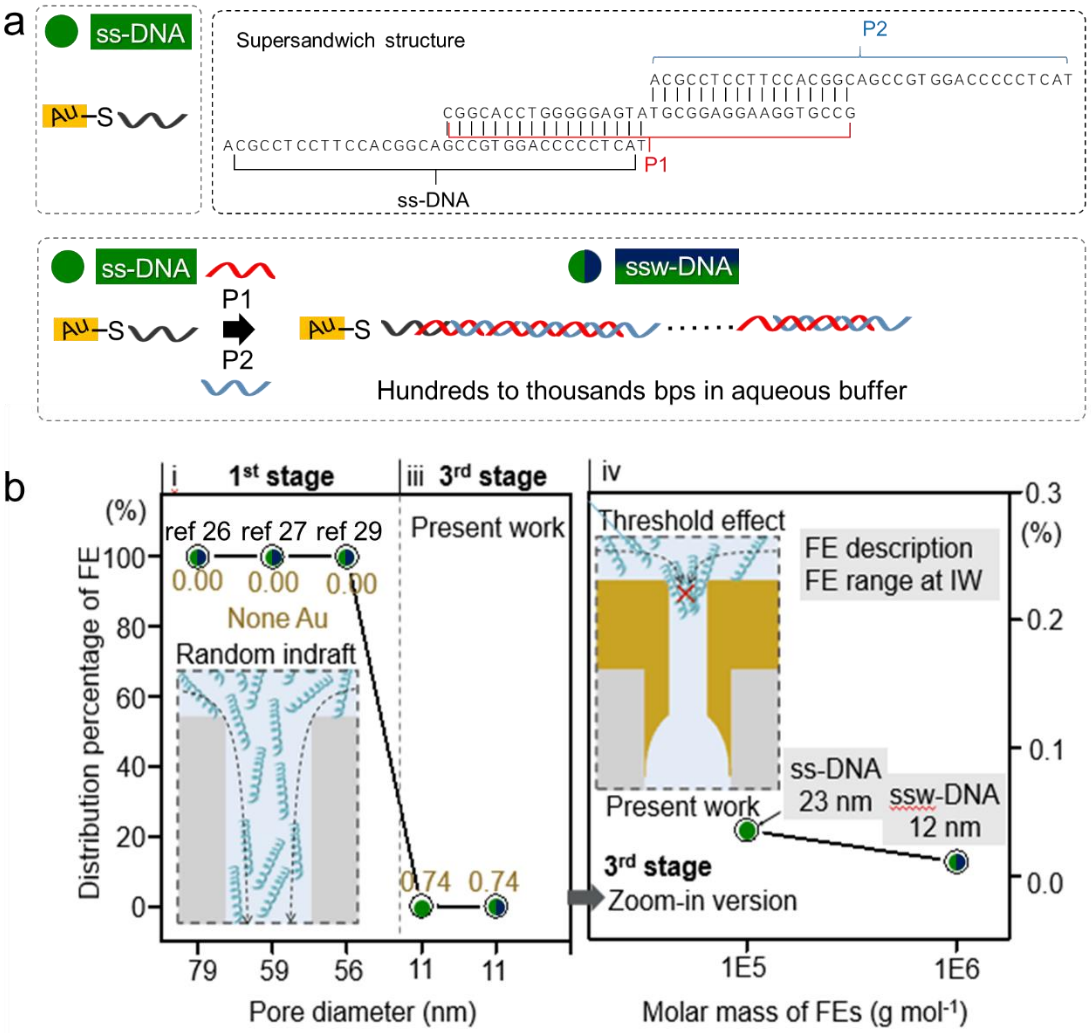

Supplementary Fig. 29. Comparison of the distribution percentage of ssw-DNA at IW in the 1st and 3rd stage. "supersandwich" DNA, a DNA amplificated structure, were used as FE in (i) 1st and (iii) 3rd stage. **a,** the scheme of ssw-DNA, in which the ss-DNA has the same sequence with the one part of ssw-DNA (P2). The different between ss-DNA and P2 is ss-DNA modified with thiol-group. The length of ssw-DNA is greater than the length of ss-DNA. **b,** in the 1st stage, the ssw-DNA occupy the total depth of IW (≈ 100%) through the random indraft of FE (the inset). In the 3rd stage, the distribution percentage of ssDNA and sswDNA at IW sharply decline near zero. In the (iii) zoom-in version (iv), the depth of ssw-DNA in nanochannels is down to 12 nm, less than the depth of ss-DNA.

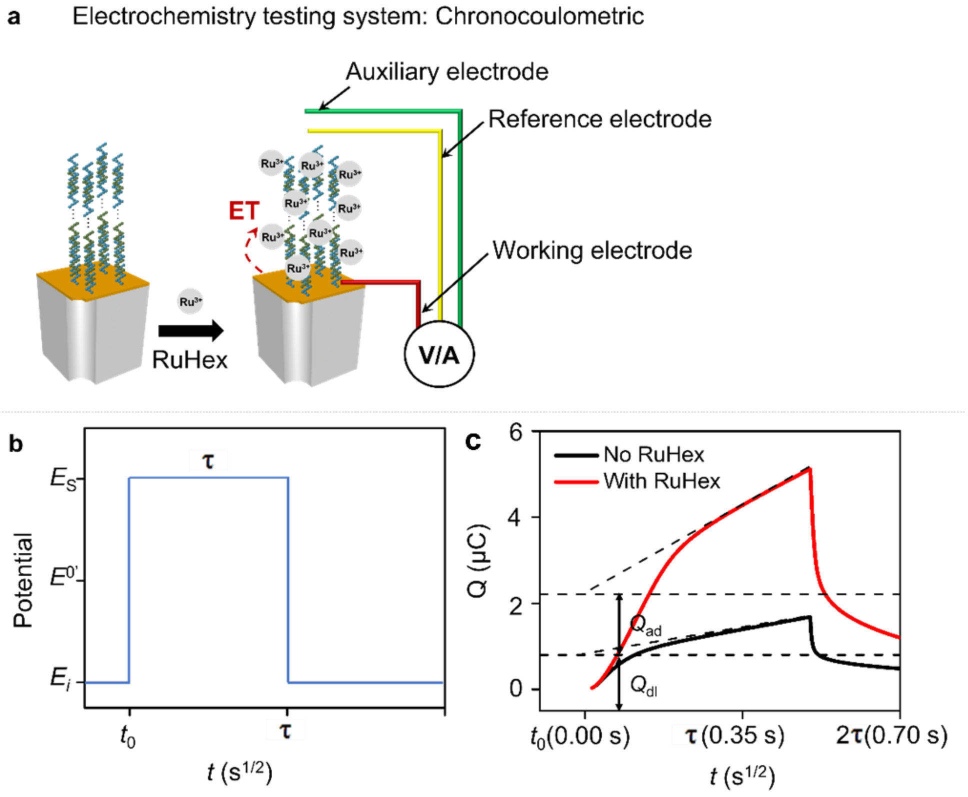

Supplementary Fig. 30. Chronocoulometric quantitation of surface charge density of OS attached with sswDNA. Chronocoulometry (CC) is employed to quantify the surface charge of sswDNA strands immobilized at OS as previously reported[3]. **a,** the scheme of three-electrode cell system. **b**, the potential versus time during the CC test. **c**, CC response curves for the OS attached with sswDNA in the absence (black) and presence (red) of 50 μM RuHex. Plot charge (*Q*) versus $t^{1/2}$ and obtain the charge for the sswDNA-modified OS (Q) from the intercept at t = 0. Based on this method, we can estimate the change of OS' charge with the treatment of ATP.

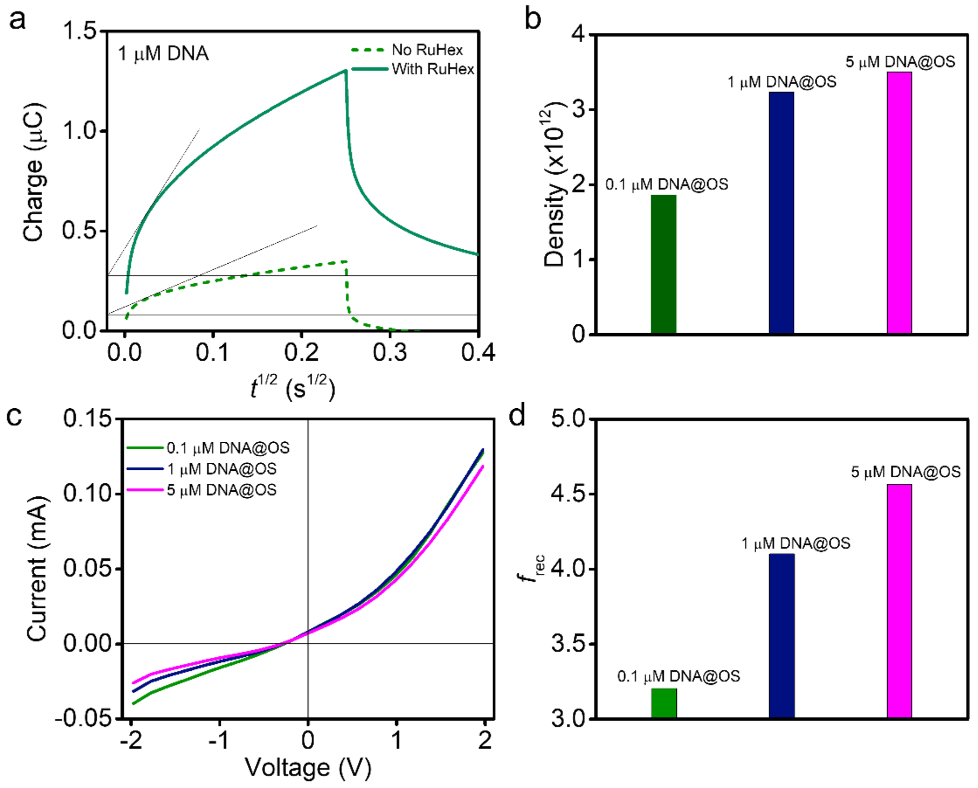

Supplementary Fig. 31. The effect of DNA grafting density on the ion transport. (**a** and **b**), the electrochemical characterization of different DNA grafting density at outer surface and (**c** and **d**), the effect of DNA with different grafting density on the ion current rectification ratio.

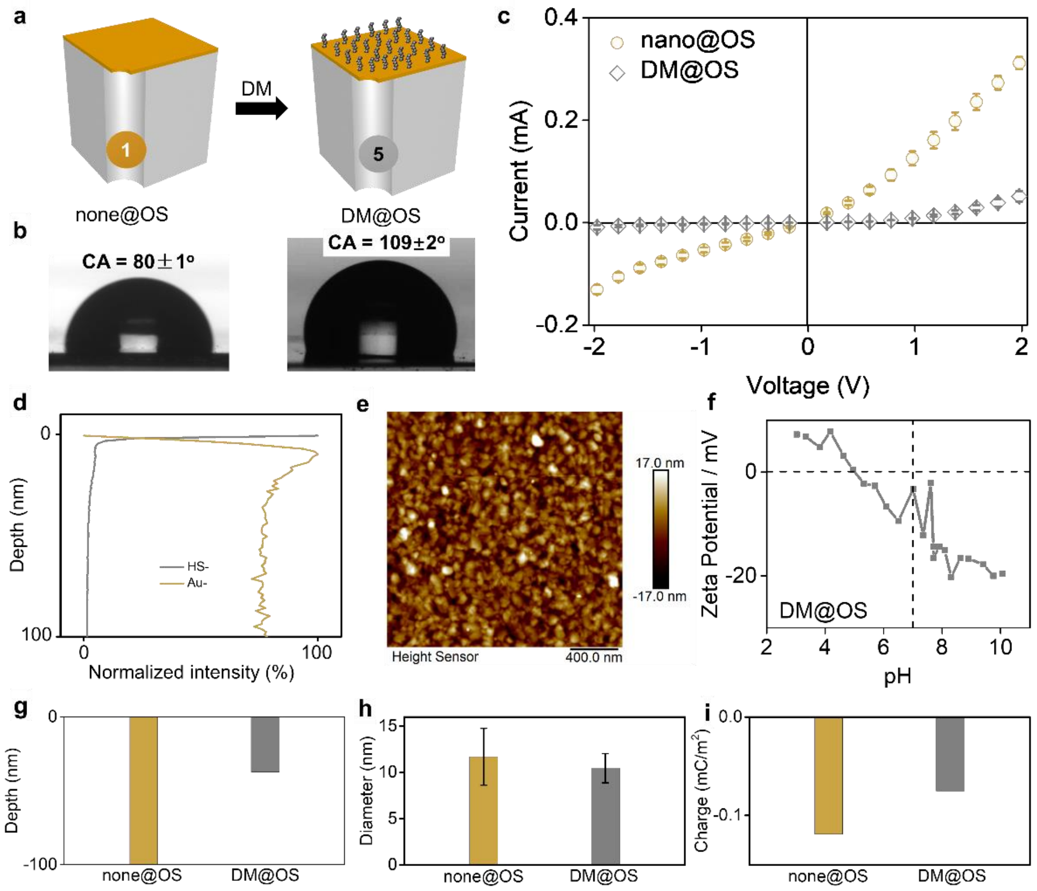

Supplementary Fig. 32. The ion transport properties of nanochannels with hydrophobic molecules (DM) at OS. a, Sketch map of the functionalization of OS with DM, named DM@OS. **b**, After the functionalization of OS with DM, the water contact angle obviously increased from 80° to 110°. **c**, *I-V* curves of none@OS and DM@OS. Error bars represent the standard deviation of calculations of 5 samples at least. After functionalization, the current obviously decreased while the f_{rec} increased. **(d, e, f)** The results come from the simulation assigned the parameter measured by ToF-SIMS, AFM, and SSZPA. **(g, h, i)** The corresponding DM distribution along the nanochannel, diameter and surface charge density of DM@OS.

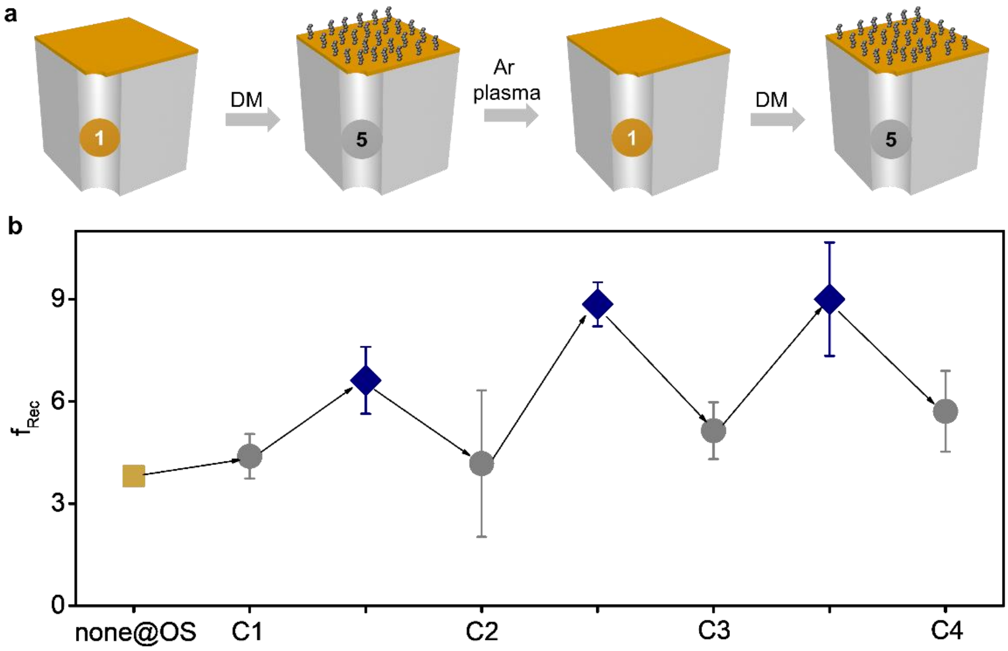

Supplementary Fig. 33. The reuse properties of nanochannel. a, The strategy of preparing the reused nanochannel. **b**, The ion transport properties of the reused nanochannels. Error bars represent the standard deviation of calculations of 3 samples at least.

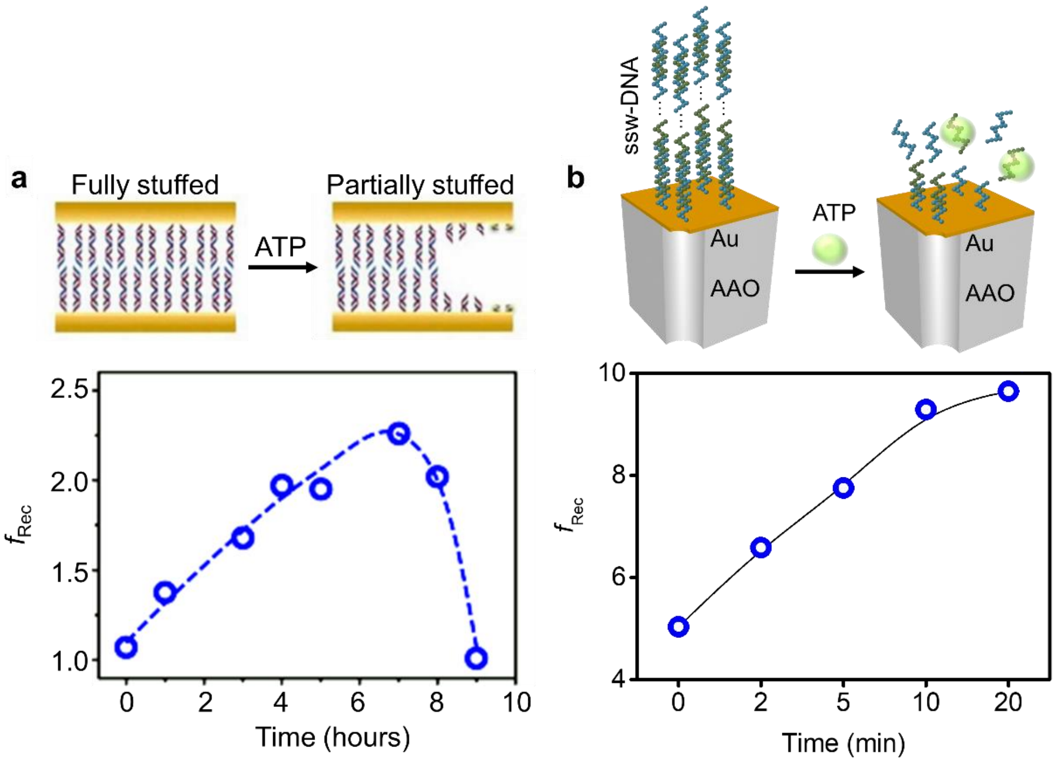

Supplementary Fig. 34. The contract of reaction dynimic of nanochannel with FE_{OS} and FE_{IW}. a, Time evolution of the dynamic ICR effect of nanochannel with FE_{IW} in previous report[3]. **b**, Time evolution of the dynamic ICR effect of nanochannel with FE_{OS} in present work. It was noted that the ssw-DNA sequences in present work is the same as the reference. And the disassembly of ssw-DNA is triggered by ATP in the present work and the reference. The difference between the two works is that the disassembly of ssw-DNA took place at the inner wall of nanochannel in the reference and at the outer surface of nanochannel in the present work.

2. Supplementary Tables

Supplementary Table S1. The calculated area of IW and OS in previous reports.

	Diameter (cm)	Pore density	Thickness (cm)	The area of IW (cm^2)	The area of OS (cm^2)
Ref39[4]	3.50E-06	2.10E+10	4.00E-03	923.16	0.80
Ref38[5]	2.00E-05	1.00E+09	6.00E-03	376.80	0.69
Ref40[6]	1.00E-05	4.00E+09	6.00E-03	753.60	0.69
Ref27[7]	5.60E-06	6.60E+09	6.00E-03	696.33	0.84
Ref29[8]	6.00E-06	6.60E+09	8.00E-03	994.75	0.81

Supplementary Table S2. The DNA sequences to prepare the ssw-DNA structure.

	**Sequence**
ss-DNA	5'-SH-(CH_2)6-CGGCACCTGGGGGAGTATTGCGGAGGAAGGTGCCG-3'
P1	5'-TACTCCCCCAGGTGCCGACGGCACCTTCCTCCGCA-3'
P1-Cy5	5'-TACTCCCCCAGGTGCCGACGGCACCTTCCTCCGCA (Cy5)-3'
P2	5'-CGGCACCTGGGGGAGTATTGCGGAGGAAGGTGCCG-3'

Supplementary Table S3. DNA aptamer sequences for the detection of targets.

	**Probe sequence**
Hg^{2+}[9]	5'-SH-(CH_2)$_6$-CCTCTCTTTCTCCCCTGTTTGTGT-3'
ATP	5'-SH-(CH_2)$_6$-CGGCACCTGGGGGAGTATTGCGGAGGAAGGTGCCG-3'
Lysozyme[10]	5'-SH-(CH_2)$_6$-ATCTACGAATTCATCAGGGCTAAAGAGTGCAGAGTTACTTAG -3'
MCF-7 Cells[11]	5'-SH-(CH_2)$_6$-GCAGTTGATCCTTTGGATACCCTGG-3'

Supplementary Table S4. Boundary conditions for the numerical solution.

Surface	Nernst-Planck Equation<br>eq 1	Poisson Equation<br>eq 2
1	$c(K^+) = c(Cl^-) = c_0$	V_b V
2	$c(K^+) = c(Cl^-) = c_0$	0 V
3，4，5，6	$\vec{n} \cdot \vec{J_\iota} = 0$	Zero charge
7, 8, 9, 10, 11, 12, 13, 14	$\vec{n} \cdot \vec{J_\iota} = 0$	$\vec{n} \cdot \nabla\varphi = -\frac{\sigma_s}{\varepsilon}$

References for Supplementary Information

1. Li, X. et al. Role of outer surface probes for regulating ion gating of nanochannels. *Nat. Commun.* **9**, 40 (2018).

2. Kim, D., Duan, C., Chen, Y. & Majumdar, A. Power generation from concentration gradient by reverse electrodialysis in ion-selective nanochannels. *Microfluid. Nanofluid.* **9**, 1215-1224 (2010).

3. Steel, A. B., Herne, T. M. & Tarlov, M. J. Electrochemical quantitation of DNA immobilized on gold. *Anal. Chem.* **70**, 4670-4677 (1998).

4. Lee, S. B. Antibody-based bio-nanotube membranes for enantiomeric drug separations. *Science* **296**, 2198-2200 (2002).

5. Wang, X. & Smirnov, S. Label-free DNA sensor based on surface charge modulated ionic conductance. *ACS Nano* **3**, 1004-1010 (2009).

6. Jiang, Y., Liu, N., Guo, W., Xia, F. & Jiang, L. Highly-efficient gating of solid-state nanochannels by DNA supersandwich structure containing ATP aptamers: a nanofluidic implication logic device. *J. Am. Chem. Soc.* **134**, 15395-15401 (2012).

7. Zhou, Y. et al. High-temperature gating of solid-state nanopores with thermo-responsive macromolecular nanoactuators in ionic liquids. *Adv. Mater.* **24**, 962-967 (2012).

8. Jiang, Y. et al. On the origin of ionic rectification in DNA-stuffed nanopores: the breaking and retrieving symmetry. *J. Am. Chem. Soc.* **139**, 18739-18746 (2017).

9. Zhang, Y., Gao, L., Wen, L., Heng, L. & Song, Y. Highly sensitive, selective and reusable mercury(ii) ion sensor based on a ssDNA-functionalized photonic crystal

film. *Phys. Chem. Chem. Phys.* **15**, 11943-11949 (2013).

10. Ali, M., Nasir, S. & Ensinger, W. Bioconjugation-induced ionic current rectification in aptamer-modified single cylindrical nanopores. *Chem Commun* **51**, 3454-3457 (2015).

11. Bian, Q., Wang, W., Wang, S. & Wang, G. Light-triggered specific cancer cell release from cyclodextrin/azobenzene and aptamer-modified substrate. *ACS Appl. Mater. Inter.* **8**, 27360-27367 (2016).
